# Prevalence of bacterial uropathogens and their antimicrobial susceptibility patterns among pregnant women in Eastern Ethiopia: hospital-based cross-sectional study

**DOI:** 10.1186/s12905-021-01439-6

**Published:** 2021-08-07

**Authors:** Alemseged Workneh Ejerssa, Diriba Alemayehu Gadisa, Teferra Abula Orjino

**Affiliations:** 1 Department of Pharmacy, Harar Health Sciences College, P.O.Box 228, Harar, Harari Regional State Ethiopia; 2 Department of Pharmacy, College of Medicine and Health Sciences, Ambo University, P.O. Box 19, Ambo, Oromia Regional State Ethiopia; 3 Department of Pharmacology, College of Health Sciences, School of Pharmacy, Addis Ababa University, P.O.Box 1176, Addis Ababa, Oromia Regional State Ethiopia

**Keywords:** UTI, Pregnant women, Bacterial uropathogens, Antimicrobial susceptibility

## Abstract

**Background:**

Urinary tract infection (UTI) is the commonest bacterial infections during pregnancy, leading to significant maternal and prenatal morbidity and mortality.

**Method:**

This hospital-based cross-sectional study during November 2017 to January 2018 was aimed to determine hospital-based antibacterial susceptibility patterns of bacterial uropathogens among 200 pregnant women in Eastern Ethiopia. ~ 10–20 ml clean-catch midstream urine samples were collected by the study participants. The well-mixed urine samples standardized to 1 µl have inoculated onto Cystine Lactose Electrolyte-Deficient and MacConkey agar. The inoculum was cultured at 37 °C under aerobic conditions for 18–48 h and examined macroscopically to evaluate the colony appearance and size of colonies. The isolate on the plates with pure growth and colonies ≥ 10^5^ CFU/ml were further subjected to biochemical identification and susceptibility testing according to the standard procedures explained in the Clinical and Laboratory Standards Institute guideline. SPSS version 25 was used for data analysis. Descriptive statistics such as frequency, percentage, and cross-tabulation were used to present the findings in the form of graphs and tables.

**Results:**

The response rate for this study was 98.04%. Thirty-one bacteria were isolated among the 200 urine samples processed, which gave the overall UTI prevalence of 15.5%. The majority (90.3%) of the isolates were Gram-negative. *Escherichia coli* (45.2%) was the most frequent isolated uropathogen which followed by *Proteus* spp. (22.6%), *Klebsiella pneumoniae* (16.1%), *Staphylococcus aureus* (9.7%), and *Pseudomonas aeruginosa* (6.5%). Among the isolates, 96.4% of them were susceptible to amikacin and followed by nitrofurantoin (90.3%), and gentamicin (83.9%). However, high rates of resistance to ampicillin (58.1%), amoxicillin-clavulanate (51.6%), and cotrimoxazole (51.6%) were observed. Overall, 16(51.6%) of the bacterial isolates had developed multiple drug resistance to the selected antimicrobials.

**Conclusion:**

In general, the overall prevalence of UTI was high, 15.5%. Most of isolated bacterial uropathogens were Gram-negative bacteria, and *Escherichia coli* was the most frequent isolate. The majority of the isolates were susceptible to amikacin, nitrofurantoin, and gentamicin. However, a high rate of resistance was observed to ampicillin, amoxicillin-clavulanate, and cotrimoxazole. More than half of the isolated bacteria had multiple drug-resistant features. Therefore, periodic and continuous urine culture for screening and diagnosis is mandatory to reduce the consequence of UTI and multidrug resistance bacteria in pregnancy.

**Supplementary Information:**

The online version contains supplementary material available at 10.1186/s12905-021-01439-6.

## Background

Urinary tract infection(UTI) is the most common infectious disease that affecting half of the population at least once during their lifetime and can lead to significant health problems [1]. It is mainly caused by Gram-negative pathogens such as *Escherichia coli*, *Proteus mirabilis*, *Klebsiella pneumoniae*, and *Enterobacter* species (spp) [2–5].

Urinary tract infection can be classified as upper UTI such as pyelonephritis (infection of the kidney) and lower UTI such as cystitis, urethritis, and prostatitis based on the affected anatomical site [6]. Moreover, it can also be grouped as complicated or uncomplicated and/or symptomatic or asymptomatic [7–9].

The prevalence of UTI is much more common in women than in men, at a ratio of 8:1, due to their anatomical and physiological reasons [10]. One in five adult women experiences UTI in her life [9, 11]. Altered physiological, anatomical, hormonal changes, and challenges in personal hygiene during pregnancy, and other factors make the antenatal mother more prone to infection of the urinary tract than nonpregnant women [12–15]. And it’s a major health problem reported among 20% of pregnant women and a common cause of admission in obstetrical wards [16].

If the infection is left untreated, it results in low birth weight, fetus, intrauterine growth retardation, preterm labor, and premature babies, intrauterine fetal death, and increased prenatal mortality and morbidity as well as maternal complications including anemia, preeclampsia, renal failure, septicemia, and adult respiratory syndrome [17]. Unlike in developed countries, its prevalence in developing countries is on the rise due to malnutrition, low socioeconomic status as well as inappropriate use of antibiotics [18]. Likely, different studies in Ethiopia indicated that the prevalence of UTI during pregnancy was ranged from 9 to 14% [19–22].

Treatment of UTI is often not started based on susceptibility tests [23]. The emergence of antibiotic resistance among urinary pathogens has been increasing worldwide [7] and it becomes a serious global public health issue [24] particularly in the developing countries where a high level of poverty, ignorance, poor hygienic practices, high prevalence of fake and spurious drugs of questionable quality in circulation is the contributing factors [25].

Since antimicrobial resistance is varied regionally, it is necessary to know the distribution of urinary pathogens and their susceptibility to antibiotics in a particular setting [26] that could support the most effective empirical treatment [27]. Moreover, antimicrobial resistance is an evolving process that needs routine surveillance and monitoring studies [4].

Due to the limited microbiology laboratory setup, routine culture and antibiotic susceptibility testing are not performed; the treatment of UTI in Ethiopia is on an empirical basis. Physicians in the study area prescribe different drugs without the guidance of culture and antibiotic susceptibility tests to treat patients with a presumptive diagnosis of UTI, which could lead to the overuse of antibiotics and the development of resistant microbial species. However, in Ethiopia, particularly in eastern Ethiopia, there is a scarcity of recent data that indicate the magnitude of the problem among pregnant women.

For the rational use of the existing antimicrobials available, a piece of up-to-date information on antimicrobial resistance needs to be available at all levels. Therefore, this study was carried out to determine the most prevalent uropathogens that caused UTI and their antimicrobial susceptibility pattern among pregnant women attending antenatal care (ANC) at Hiwot Fana Specialized University Hospital (HFSUH), Harar, Eastern Ethiopia.

## Methods and patients

### Study area, design, period, and patients

The present study was undertaken at antenatal care clinics of HFSUH which is affiliated with Haramaya University College of Medicine and Health Sciences. It is found in the Harari Regional State, Harar, Eastern Ethiopia which is found 525 km from the capital city, Addis Ababa.

A hospital-based cross-sectional study design was conducted from November 2017 to January 2018. All pregnant women who had a follow-up and attended the antenatal care clinic of HFSUH, and willing to participate in the study during the study period were consecutively recruited. However, pregnant women who received antibiotics within 15 days before ANC follow-up and who were treated for another infection were excluded from the study.

### Sample size determination and sampling technique

A single population formula was used to calculate the sample size using the following parameter, a 14% prevalence rate of UTI among pregnant women attending the antenatal clinic of Dil Chora Referral Hospital, Dire Dewa Ethiopia [21]. Tolerable margin of error (d) = 5%, Z score for 95% confidence interval, with 10% non-response rate.

$$ {\text{n}} = \frac{{{\text{z}}2{\text{ p }}\left( {1 - {\text{p}}} \right)}}{{{\text{d}}2}} $$

where z = Z score for 95% confidence interval, a = 1.96, P = prevalence, d = margin of error (5%).m; n = $\frac{{{\text{z}}2{\text{ p }}\left( {1 - {\text{p}}} \right)}}{{{\text{d}}2}}$ = $\frac{{1.962{*}0.14{ }\left( {1 - 0.14} \right)}}{{0.05{*}0.05}}$ = 185; Non-response rate = 185 ~ 10% = 18.5 ~ 19; Total sample size was = 185 + 19 = 204.

All pregnant women attending the antenatal clinics of HFSUH and that fulfilled the inclusion criteria till the required sample size attained were selected using a convenient sampling technique.

### Data collection procedure

A semi-structured questionnaire (see Additional file 1: File 1) that was adopted from Derese et al. [21], and modified based on the study objective, was used for the collection of clinical/socio-demographic data. It demonstrates an identification number of the patient, age, pregnancy (gestational age) and marital status, urinary signs and symptoms, predisposing factors, and other related information. Culture and susceptibility results were also included in the questionnaire to record their culture and susceptibility test results.

### Urine sample collection and transportation

After face to face interview about sociodemographic and clinical data related to UTI was completed with each pregnant woman, they were sent to the hospital laboratory with their request form. Then, all study participants requested to bring ∼10–20 mL clean-catch midstream urine specimens after urine sample collection techniques were explained by trained medical laboratory professionals. Accordingly, all study participants have thoroughly washed their hands with water and drying. And they told to separate their labial by one hand for cleaning around the urinary opening area with water in the backward direction and dry thoroughly. Then, by separating the labia, the first 20–30 ml urine was voided in the toilet, and ∼10–20 mL clean- catch midstream urine samples were collected into a sterile universal utensil. Finally, they closed the cap of the urine bottle immediately considering not to touch either the edge of the bottle or the inner side of the bottle cap. The bottle was labeled with the unique client’s identification (ID) number, date, and collection time by medical laboratory professionals.

**Fig. 1:**
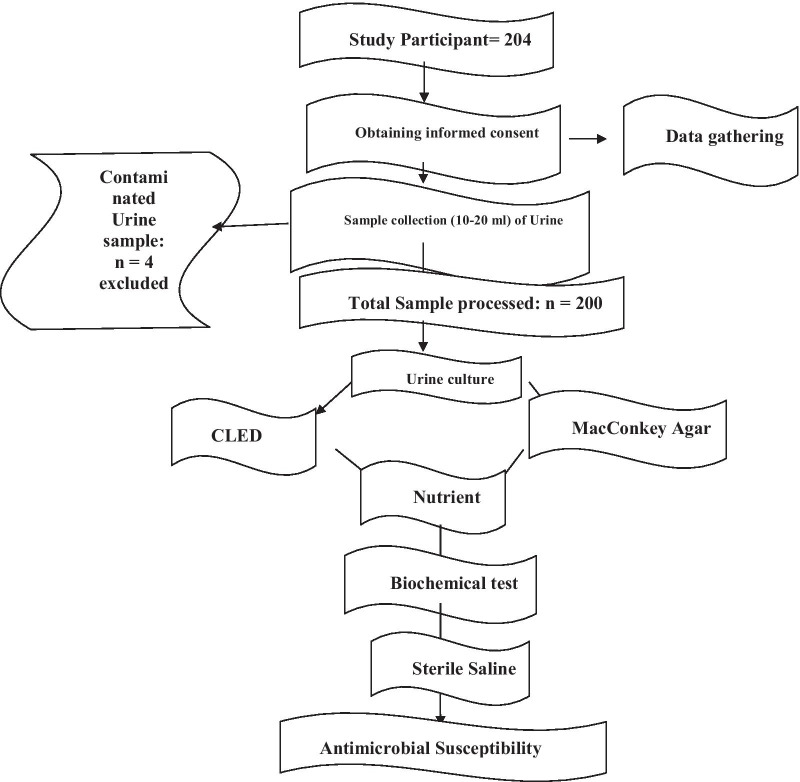
Data collection and sample processing flow chart

We could not conduct urine sample processing immediately within 30 min of its collection since sample collection and urine processing for culture were not conducted at the same site. Hence, to maintain the quality of the samples collected, we stored them in the refrigerator of the central medical laboratory of HFSUH until we sent all together within two hours of its collection to the microbiology department of Harari Health Research and Regional Laboratory which was around 150 m away from the study area. The samples were stored in the cold box during transportation to the microbiology department. Then, once each sample reach the microbiology department, it was processed immediately for culture. Four samples were excluded (i.e.1 contaminated with stool and 3 not refrigerated properly) while the rest 200 samples were processed for culture after proper mixing. For more, please see Fig. 1.

### Bacterial isolation and identification

Once each urine sample was delivered to the microbiology laboratory; it screened for the presence of bacterial agents according to the standard procedures for the diagnosis of bacterial UTI and isolation/identification of bacteria from urine [28]. Hence, the well-mixed, as well as non-centrifuged specimens, were inoculated using a standard wire loop that can deliver 1µL of urine specimen onto Cystine Lactose Electrolyte-Deficient (CLED) and MacConkey agar (with and without crystal violet, Titan, Biotech LTD, India) through a surface streak procedure. CLED was used because it gives consistent results and allows the growth of both Gram-negative and Gram-positive bacterial pathogens.

The plates were put in an incubator at 37 °C overnight (18–24 h) under aerobic conditions. After 24 h of incubation, the plates were examined for the presence or absence of the bacterial growth macroscopically (unaided eyes). Then those plates with positive cultures were examined macroscopically to evaluate the colony appearance and its size. Subculturing of not well-isolated colonies was performed using a sterile wire loop to ensure pure cultures. Then, the confirmed colonies were counted from CLED and multiplied by 1000 to determine the number of bacteria per milliliter (CFU/ml) of the original urine specimen. Plates with significant bacteria, pure growth, and colonies ≥ 10^5^ CFU/ml, were further subjected to identification and susceptibility testing.

A cut point of ≥ 10^5^ CFU/ml was used to define UTI. The bacterial isolates were identified using standard biochemical tests [29, 30]. Accordingly, Gram-negative bacteria were identified by performing a series of biochemical tests, namely oxidase, indole, Simmons citrate agar(citrate utilization), lysine decarboxylation, lactose fermentation, gas, and Hydrogen Sulfide (H_2_S) production as well as motility tests. Gram-positive bacteria were also identified based on their catalase test and coagulase tests. After identification of the specific bacteria was completed, the antimicrobial susceptibility test of isolated pathogens was followed ensuring that appropriate and adequate antibiotics were provided.

### Antimicrobial susceptibility testing (AST)

Antibiotic susceptibility testing was done by using Kirby Bauer (disk diffusion) method on Muller Hinton agar according to the standard procedures [29, 30]. The suspension of the bacterium was prepared by picking a pure colony with a sterile wire loop, suspended and emulsified into a test tube containing 5 ml of nutrient saline, and then mixed gently until the uniform suspension was formed. Standard inoculums were adjusted to 0.5 McFarland which yield a uniform suspension containing 10^5^–10^6^ cells/mL.

Using sterile applicator cotton-swab, a sample of the standardized inoculum was taken and streaked back and forth on the entire surface of the dried Mueller–Hinton agar plate (Biomark, Laboratory, India). The streaking procedures were repeated and the plates were turned at a 60° angle between each streaking to ensure even distribution and then the inoculums were allowed to dry for 5–15 min with the lid in place. Then, using sterile forceps, the selected antibiotics disks were applied to the plates. The antimicrobial agents used for susceptibility testing were: augmentin, (AMC, 30 µg), ampicillin (AMP, 10 µg), chloramphenicol (C, 30 µg), ciprofloxacin (CIP, 5 µg), norfloxacin (NOR, 10 µg), trimethoprim + sulphamethazole (SXT, 25 µg, 1.25/23.75 µg), amikacin(AK, 10 µg), gentamicin (GEN, 10 µg), ceftriaxone (CRO, 30 µg), ceftazidime (CAZ, 30 µg), nalixidic acid (NA, 30 µg), and nitrofurantoin (F, 300 µg). All antimicrobials used for the study were Oxoid Ltd. Bashing store Hampshire, UK products.

The discs were placed at least 24 mm away from each other and 15 mm from the edge to avoid the overlapping of the zone of inhibition and pressed down to ensure complete contact with the agar surface. The plates were inverted upside down and incubated aerobically at 37 °C for 18–24 h. The diameter of the zone of inhibition around each disc was measured to the nearest wholly millimeter (mm) from the back of the plate by using reflected light and a digital caliper. Then, the bacterial isolates were classified as susceptible (S), intermediate (I), or resistance (R) by comparing them against the zone of inhibition diameter of interpretative standards as indicated in the Clinical and Laboratory Standards Institute (CLSI) guideline [29, 31] (For more see Additional file 2: File 2). Bacterial isolates resistant to three or more antimicrobials belonging to different structural classes were classified as MDR [32]. The overall procedure layout is depicted in Fig. 1.

### Quality control

The semi-structured questionnaire was first prepared in English language and translated to local Amharic language, back-translated to English to ascertain its consistency, and pretested on 5% of the sample size at HFSUH. Before the actual work commenced, practical refreshment training was given to data collectors (i.e. medical laboratory technicians) on the aim of the study and the methodology part regarding urine sample collection and processing for bacterial isolation, identification, and AST for half-day by investigators.

Participants were oriented on how to collect self-midstream urine samples by trained data collectors. The specimens were stored in the refrigerator of HFSUH Central Medical laboratory with controlled temperature, and transported to the Harari Health Research Center and regional laboratory within 2 h of its collection in the cold box, and immediately processed by the Medical Microbiologist. Each culture media was checked daily to observe crack contamination, and decolorization formed during the culture process. Standard operating procedures and the manufacturer’s instruction manual were strictly followed for isolation and identification, and the drug susceptibility testing of the isolated pathogen. Moreover, the reference strains such as *Escherichia coli* (American Type Culture Collection (ATCC) 25922), *Staphylococcus aureus* (ATCC25923), and *Pseudomonas aeruginosa* (ATTC 27853) were used as a control to check the culture performance and drug disks. All the standard strains were obtained from the Harari Health Research Center and regional laboratory.

### Data processing and analysis

The collected data were checked for completeness, coded, entered, and cleaned using Epi-data version 3.1, and was exported and analyzed using SPSS version 25 software. Descriptive statistics such as frequency, percentage, and cross-tabulation were used to present the findings in the form of a graphs and tables.

### Operational definition

- *Urinary tract infection (UTI)/significant bacteriuria* a culture that grew ≥ 10^5^colony-forming units (CFU/mL) in a single voided 10–20 ml midstream urine.
- *Symptomatic UTI* a patients whose urine is yielding positive cultures (≥ 10^5^ CFU/ml) and who have symptoms referable to the urinary tract.
- *Asymptomatic bacteriuria* significant growth of the pathogen (≥ 10^5^ bacteria/ml) in the absence of clinical manifestation.
- *Mid-stream urine specimen* a specimen obtained from the middle part of urine flow: clean catch urine specimen.
- *Multiple drug resistance* bacterial isolates resistant to three or more antimicrobials belonging to different structural classes.
- *History of UTI* is any history of infection about the urinary tract diagnosed by a physician.
- *Previous antibiotic use* patients who had received antibiotic therapy with in 15 days before enrolled in to the study.

## Results

### Socio-demographic, obstetrics, and clinical characteristics of the study participants

Among 204 samples collected, only 200 samples were processed for culture and susceptibility test which gives a 98.04% response rate. The majority, 72(36%), of the study participants, were aged between 23 and 27 years. More than 89% of the study participants lived in an urban area. Moreover, 61(30.5%) had educational level of secondary cycle (9–12 grade), 185 (92.5%) married, and 61(30.5%) were civil servant. Their obstetric data showed that 79% had gravidity of 1–3 times. Among the study participants, 27% had a history of UTI, 6.5% underwent obstetric and gynecologic surgery, and 4% reported prior use of an indwelling catheter (Table 1).

**Table 1:** Socio-demographic, obstetric, and clinical variables of pregnant women attended the ANC clinic of HFSUH, Harar, Eastern Ethiopia, from November 2017 to January 2018

Patient characteristics	Total participant (n = 200)	
	Frequency (n)	Percentage (%)
Age (in the year)		
18–22	52	26
23–27	72	36
28–32	48	24
33–37	15	7.5
38–42	12	6
≥ 43	1	0.5
Residence		
Rural	22	11
Urban	178	89
Educational status		
Cannot read and write	28	14
Had informal education	33	16.5
Primary cycle(1–8)	46	23
Secondary cycle(9–12)	61	30.5
Higher education(> 12)	32	16
Marital status		
Married	185	92.5
Divorced	10	5
Widowed	5	2.5
Gravidity		
1–3	158	79
4–6	32	16
7–9	10	5
Parity		
Nulliparous	85	42.5
Primapara(one)	50	25.0
Multipara	65	32.5
Gestational age		
1st trimester	34	17
2nd trimester	92	46
3rd trimester	74	37
History of obstetric and gynecologic surgery		
Yes	13	6.5
No	187	93.5
History of catheterization		
Yes	8	4
No	192	96
History of UTI		
Yes	54	27
No	146	73
A symptom of UTI on presentation		
Symptomatic	54	27
Asymptomatic	146	73
Diabetes mellitus		
Yes	8	4
No	192	96

ANC, antenatal care; HFSUH, Hiwot Fana Specialized University Hospital; UTI, urinary tract infection

### Prevalence of UTI across socio-demographic, obstetric, and clinical variables of pregnant women

Among the 200 urine samples investigated, 31 of them grew bacteria in culture with significant bacterial growth, which gives an overall prevalence of 15.5%. A higher rate of UTI, 45.2%, was reported among age groups 23–27 years and followed by 32.5% among the age group of 28–32 years. Among the study participants with significant bacteriuria, 27/31(87%) lived in an urban area. Those study participants, who had a history of catheterization, prior Obstetric and Gynecologic Surgery and history of UTI showed that 4/8(50%), 5/13(38.5%), and 19/54(35.2%) prevalence of UTI respectively. Out of the 200 midstream urine samples processed, 13/62(20.9%) and 18/138(13%) had shown significant bacteriuria among symptomatic and asymptomatic participants respectively. Unfortunately, among 31 study participants with positive urine culture (i.e. significant bacteriuria), 18(58.1%) of them did not complain of any symptom of UTI on presentation (see Table 2).

**Table 2:** Prevalence of UTI across socio-demographic, obstetric, and clinical variables of pregnant women attended ANC clinic of HFSUH, Harar, Eastern Ethiopia, from November 2017 to January 2018

Variables	Significant bacteriuria, n = 31 (%)	Non-significant bacteriuria, n = 169 (%)	Total (n = 200)
Age (in year)			
18–22	4(12.9)	48(28.4)	52
23–27	14(45.2)	58(34.3)	72
28–32	10(32.3)	38(22.5)	48
33–37	2(6.4)	13(7.7)	15
38–42	0(0)	12(7.1)	12
≥ 43	1(3.2)	0(0)	1
Residence			
Rural	4(13)	18(10.7)	178
Urban	27(87)	151(89.3)	22
Educational status			
Illiterate	5(16)	23(13.6)	28
Read and write	10(32.2)	23(13.6)	33
Primary cycle(1–8 grade)	7(22.6)	39(23)	46
Secondary cycle(9–12 grade)	7(22.6)	54(31.9)	61
Higher education(> 12 grade)	2(6.6)	30(17.6)	32
Marital status			
Married	30(96.8)	155(91.7)	185
Divorced	1(3.2)	9(5.3)	10
Widowed	0(0)	5(3)	5
Gravidity			
1–3	30(96.8)	128(75.7)	158
4–6	1(3.2)	31(18.3)	32
7–9	0(0)	10(6)	10
Parity			
Nulliparous	12(38.7)	73(43.2)	85
Primapara(one)	11(35.5)	39(23.1)	50
Multipara	8(25.8)	57(33.7)	65
Gestational age			
1st trimester	5(16.1)	29(17.2)	34
2nd trimester	16(51.6)	76(44.9)	92
3rd trimester	10(32.3)	64(37.9)	74
History of obstetric and gynecologic surgery			
Yes	5(16.1)	8(4.7)	13
No	26(83.9)	161(95.3)	187
History of catheterization			
Yes	4(12.9)	4(2.4)	8
No	27(87.1)	165(97.6)	192
History of UTI			
Yes	19(61.3)	35(20.7)	54
No	12(38.7)	134(79.3)	146
A symptom of UTI on presentation			
Symptomatic	13(41.9)	49(29)	62
Asymptomatic	18(58.1)	120(71)	138
Diabetes mellitus			
No	28(90.3)	164(97)	192
Yes	3(9.7)	5(3)	8

ANC, antenatal care; HFSUH, Hiwot Fana Specialized University Hospital; UTI, urinary tract infection

### Isolated bacterial uropathogens

**Fig. 2:**
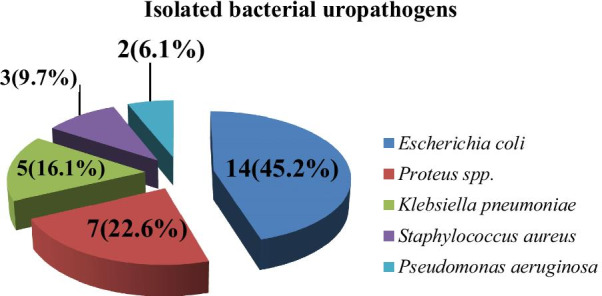
Isolated bacterial uropathogens among pregnant women with UTI attending at HFSUH, Harar, Eastern Ethiopia, from November 2017- January 2018

In our study, out of the total midstream urine sample culture test, a total of (n = 31) bacteria were isolated with five different species. Most of the isolated bacteria were Gram-negative organisms 28(90.3%) while only 3(9.7%) were Gram-positive *Staphylococcus aureus*. *Escherichia coli* was the most frequently isolated bacteria 14(45.2%) that followed by *Proteus* spp.7(22.6%), *Klebsiella pneumoniae* 5(16.1%), *Staphylococcus aureus* 3(9.7%), and *Pseudomonas aeruginosa* 2(6.4%) (see Fig. 2).

### Antimicrobial susceptibility pattern of isolated bacterial uropathogens

**Fig. 3:**
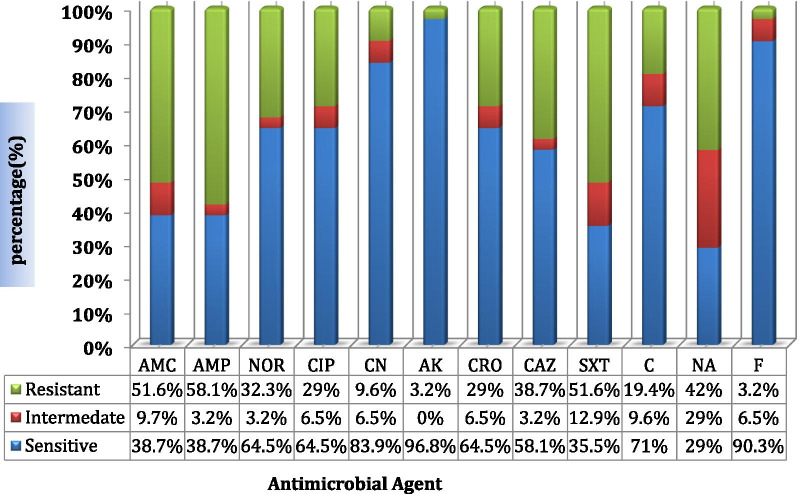
Overall antimicrobial susceptibility patterns of isolated bacterial uropathogens from the urine of pregnant women at ANC clinic of HFSUH, Harar, Eastern Ethiopia from November 2017 to January 2018. Abbreviation: AMC: amoxicillin-clavulanate, AMP: ampicillin, NOR: norfloxacin, CIP: ciprofloxacin CN: gentamicin AK: amikacin CRO: ceftriaxone CAZ: ceftazidime SXT: cotrimoxazole C: chloramphenicol NA: nalixidic acid F: nitrofurantoin

Among the identified bacterial uropathogens, 30 (96.8%) of them were susceptible to amikacin, 28(90.3%) were susceptible to nitrofurantoin, 26(83.9%) were susceptible to gentamicin, and 22(71%) showed susceptibility to chloramphenicol. However, high rates of resistance were observed to ampicillin 18(58.1%), amoxicillin-clavulanate 15(51.6%), cotrimoxazole 15(51.6%), and nalixidic acid 13(42.0%). For more see Table 3 and Fig. 3.

**Table 3:** Antimicrobial susceptibility patterns of bacteria isolated from the urine of pregnant women at ANC clinic of HFSUH, Harar, Eastern Ethiopia, from November 2017 to January 2018

Isolated bacteria	Total	Pattern	Antimicrobial agents tested											
			AMC n (%)	AMP n (%)	NOR n (%)	CIP n (%)	CN n (%)	AK n (%)	CRO n (%)	CAZ n (%)	SXT n (%)	C n (%)	NA n (%)	F n (%)
*Escherichia coli*	14	S I R	8(57.1) – 6(42.9)	7(50) – 7(50)	10(71.4) – 4(28.6)	10(71.4) – 4(28.6)	13(92.3) – 1(7.7)	14(100) – –	10(71.4) 1(7.1) 3(21.5)	10(71.4) 1(7.13) 3(21.5)	6(42.9) 2(14.2) 6(42.9)	10(71.4) 2(14.3) 2(14.3)	6(42.9) 3(21.5) 5(3.6)	12(85.7) 2(14.3) –
*Proteus* spp.	7	S I R	1(14.3) 2(28.6) 4(57.1)	2(28.6) 1(14.3) 4(57.1)	4(57.1) 1(14.3) 2(28.6)	4(57.1) 1(14.3) 2(28.6)	5(71.4) 2(28.6) –	7(100) – –	3(42.9) 1(14.2) 3(42.9)	3(42.9) – 4(57.1)	1(14.3) 1(14.3) 5(71.4)	4(57.1) – 3(42.9)	1(14.3) 1(14.3) 5(71.4)	7(100) – –
*Klebsiella pneumoniae*	5	S I R	1(20) 1(20) 3(60)	1(20) – 4(80)	3(60) – 2(40)	3(60) 1(20) 1(20)	4(80) – 1(20)	5(100) – –	3(60) – 2(40)	3(60) – 2(40)	3(60) 1(20) 1(20)	5(100) – –	1(20) 2(40) 2(40)	5(100) – –
*Pseudomonas aeruginosa*	2	S I R	– – 2(100)	– – 2(100)	1(50) – 1(50)	1(50) – 1(50)	1(50) – 1(50)	1(50) – 1(50)	2(100) – –	1(50) – 1(50)	1(50) – 1(50)	1(50) – 1(50)	0(0) 1(50) 1(50)	1(50) – 1(50)
*Staphylococcus aureus*	3	S I R	2(66.7) – 1(33.3)	2(66.7) – 1(33.3)	2(66.7) – 1(33.3)	2(66.7) – 1(33.3)	3(100) – –	3(100) – –	2(66.7) – 1(33.3)	1(33.3) – 2(66.6)	1(33.3) – 2(66.7)	2(66.7) 1(33.3 –	1(33.3) 2(66.7) –	3(100) – –
Total		S I R	12(38.7) 3(9.7) 15(51.6)	12(38.7) 1(3.2) 18(58.1)	20(64.5) 1(3.2) 10(32.3)	20(64.5) 2(6.5) 9(29)	26(83.9) 2(6.5) 3(9.7)	30(96.8) – 1(3.2)	20(64.5) 2(6.5) 9(29)	18(58.1) 1(3.2) 12(38.7)	11(35.5) 4(12.9) 16(51.6)	22(71) 3(9.7) 6(19.4)	9(29) 9(29) 13(42)	28(9.1) 2(6.5) 1(3.2)

AMC, amoxicillin-clavulanate; AMP, ampicillin; NOR, norfloxacin; CIP, ciprofloxacin; CN, gentamicin; AK, amikacin; CRO, ceftriaxone; CAZ, ceftazidime; SXT, cotrimoxazole; C, chloramphenicol; NA, nalixidic acid; F, nitrofurantoin; ‘–’, denotes 0(0)

Concerning specific isolate, all isolated *Escherichia coli* (100%) were susceptible to amikacin. Besides, 13(92.3%) and 12(85.7%) of *Escherichia coli* were also susceptible to gentamicin and nitrofurantoin respectively. However, 7(50%) of isolated *Escherichia coli* showed resistance to ampicillin, while 6(42.9%) of them developed resistance to each amoxicillin-clavulanate and co-trimoxazole.

All *Proteus* spp. 7(100%) isolated were susceptible to amikacin and nitrofurantoin while 5(71.4%) of them were susceptible to gentamicin. On the contrary, 5(71.4%) of the *Proteus* spp. were resistant to each cotrimoxazole and nalixidic acid. In addition, all isolates of *Klebsiella pneumoniae* also showed susceptibility to amikacin, gentamicin, and nitrofurantoin though still majority of them were resistant to ampicillin 4(80%) and amoxicillin-clavulanate 3(60%). The two (100%) *Pseudomonas aeruginosa* identified were also resistant to each ampicillin and amoxicillin-clavulanate. However, all of them showed susceptibility to ceftriaxone.

Similar to the majorities of identified Gram-negative bacteria, the only Gram-positive *Staphylococcus aureus,* 3(100%), were also susceptible to kanamicin, gentamicin, and nitrofurantoin. Two (66.6%) of the *Staphylococcus aureus* were showed resistance to each co-trimoxazole and ceftazidime (see Table 3**).**

### Multiple drug resistance patterns of bacterial uropathogens

**Fig. 4:**
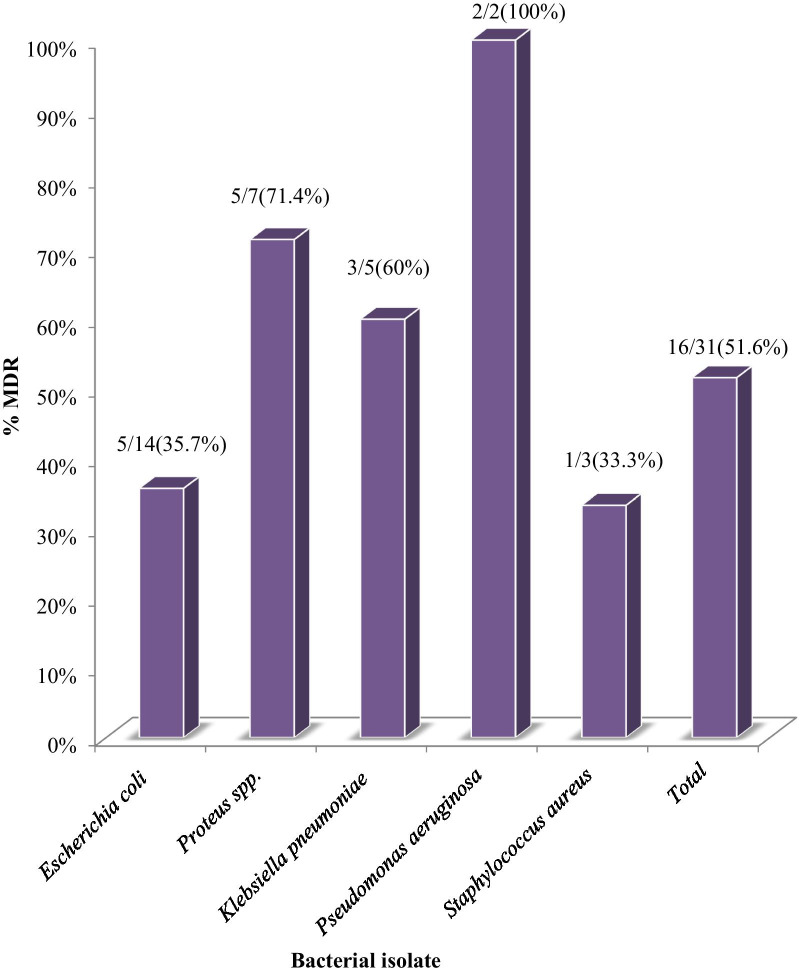
Multidrug resistance patterns of isolated bacterial uropathogens among pregnant women at the ANC clinic of HFSUH, Harar, Eastern Ethiopia from November 2017 to January 2018

Among the total isolates (n = 31), multi-drug resistance was observed in 16 (51.6%) of all bacterial uropathogens. Conversely, 2(100%) of *Pseudomonas aeruginosa,* 5(71.4%) of *Proteus* spp, and 3(60%) of *Klebsiella pneumoniae* were developed multi-drug resistance. In general, MDR features are presented in Fig. 4.

Among the isolated bacteria, 6(19.4%) of them were resistant to three structurally different antimicrobials while 5(16.1%), 4(12.9%), and 1(3.2%) of them were resistant to four, five, and six structurally different antimicrobials respectively. Only 8 (25.8%) of the bacterial isolates were susceptible to all antibiotics (see Table 4).

**Table 4:** Multidrug resistance patterns of isolated bacterial uropathogens among pregnant women attended an ANC clinic at HFSUH, Harar, Eastern Ethiopia, from November 2017 to January 2018

Antimicrobial agents^a^	Total bacterial isolates (N = 31)^#^					Total(N = 31)(%)
	*Escherichia coli* (N = 14)	*Proteus* spp. (N = 7)	*Klebsiella pneumoniae* (N = 5)	*Pseudomonas aeruginosa* (N = 2)	*Staphylococcus aureus* (N = 3)	
R3						
AMC + CTX + NA	1	–	–	–	-	1
AMC + AMP + NA + F	-	–	–	1	–	1
AMC + AMP + CIP + SXT	1	–	–	–	–	1
AMC + CRO + CAZ + NA	–	–	1	–	–	1
AMC + AMP + NOR + CIP + SXT	–	–	1	–	–	1
NA + NOR + CIP + CRO + CAZ + SXT	–	1	–	–	–	1
Total	2	1	2	1	0	6(19.4)
R4						
AMC + AMP + CAZ + SXT + NA	–	1	–	–	–	1
AMC + AMP + NOR + CIP + CRO + SXT	–	–	–	–	1	1
AMC + AMP + NA + NOR + CIP + SXT + C	–	1	–	–	–	1
AMC + AMP + NA + NOR + CN + CRO + CAZ	–	–	1	–	–	1
AMC + AMP + NA + NOR + CIP + CRO + CAZ + SXT	1	–	–	–	–	1
Total	1	2	1	0	1	5(16.1)
R5						
AMC + NA + CRO + CAZ + SXT + C	–	1	–	–	–	1
AMC + AMP + NA + CRO + CAZ + SXT + C	–	1	–	–	–	1
AMC + AMP + NA + NOR + CIP + CRO + CAZ + SXT + C	2	–	–	–	–	2
Total	2	2	0	0	0	4(12.9)
R6 AMC + AMP + NOR + CIP + AK + CN + CAZ + SXT + C						
	–	–	–	1	–	1(3.2)

AMC, amoxicillin-clavulanate; AMP, ampicillin; NOR, norfloxacin; CIP, ciprofloxacin; CN, gentamicin; AK, amikacin; CRO, ceftriaxone; CAZ, ceftazidime; SXT, cotrimoxazole; C, chloramphenicol; NA, nalixidic acid; F, nitrofurantoin

‘–’ Denotes 0(0); ^#^R_0_ = only 8 isolates were vsusceptible to all antimicrobial agents; R_3_, R_4_, R_5_, R_6_: Resistance to three, four, five, and six antimicrobials belongs to different structural classes respectively

^a^Classes of antimicrobials are: (1) AMC/AMP; (2) CRO/CAZ; (3) NA/NOR/CIP; (4) CN/AK;(5) SXT; (6) C; (7) F

## Discussion

The current study revealed that the overall prevalence of UTI among pregnant women was 15.5%. This is comparable with the prevalence of UTI reported among pregnant from a study done in Tanzania (15.5%) [33], Khartoum, Sudan (14%) [34], and with a report in Dire Dewa, Eastern Ethiopia (14%) [21]. However, it was higher than in other regions of Ethiopia, such as 11.6% from Addis Ababa [22], 10.4% from Gondar [19], and 9.5% from Bahir Dar [20]. In contrast, it was lower than a report, 75% from Niger [35] and 20% from Saudi Arabia [36]. The observed variation might be due to differences in the standard of personal hygiene and education, sample size, and social habits [14].

The prevalence of UTI (i.e. showed significant bacteriuria) among symptomatic (n = 62) and asymptomatic pregnant women (n = 138) on presentation in our study was 21% and 13%, respectively. The occurrence of UTI cases among symptomatic study participants was in line with the previous study done in Addis Ababa [22] and Dire Dewa, Ethiopia [21] which was 20% and 17% respectively. Moreover, it also in agreement with a study done in Tanzania, 17.9% [33]. However, it was slightly higher than the study conducted elsewhere such as Makka, Saudi Arabia (12%) [36], and Gondar University Hospital, Gondar, Ethiopia (10.2%) [19]. The variation might be due to differences in the study setting (primary health care or hospital), a screening test used (Urine dipstick, microscopy, or culture), or it might be due to the involvement of a small number of pregnant with clinical manifestations of UTI cases [37].

As mentioned above, 13% of asymptomatic study participants on presentation showed a positive urine culture later. This result was in agreement with a study conducted in India [38], Sudan [34], and Tanzania [33] that were 11.6%, 14.7%, and 13%, respectively. Moreover, comparable findings were also reported from Dire Dewa, Ethiopia, 11% [21]. However, the finding was higher than the study performed in Gondar, Ethiopia (9%) [19] and Makka, Saudi Arabia (8%) [36]. The main problem with asymptomatic bacteriuria in pregnancy is that the patients are asymptomatic and unless they are screened, it can remain discrete leading to grave complications for both the mother and the fetus. Therefore, a periodic examination is needed [19, 34, 38, 39].

Among the 31 bacterial uropathogens identified in this study, most of them were Gram-negative organisms 28(90.3%) while only 3(9.7%) were Gram-positive. Our finding is in line with the studies reported from Tertiary Care Hospitals in Indian in which Gram-negative were (91.3%) and Gram-positive were (8.7%) [40]. Similar reports also observed in other studies in which Gram-negative bacteria were the most common UTI-associated pathogens with a rate of 67.5% from Gondar [19], 73.1% from Dire Dewa, Ethiopia [21], 75% from Kenyatta National Hospital, Kenya [14]. This might be due to the existence of a unique structure in Gram-negative bacteria which helps their attachment to the uroepithelial cells, multiplication, and tissue invasion resulting in invasive infection during the gestation period [34, 39].

*Escherichia coli* was the predominant (45.2%) isolate among the isolated uropathogens in our study. Different studies conducted in different parts of the globe also reported similar findings to our result with respect to *Escherichia coli* with a rate of 50% from Afikpo Ebony state, Nigeria [41], 42.7% from Chandanaish, Bangladesh [42]**,** 42.4% from Khartoum, Sudan [34]**,** and 41.5% from Yemen [43]. Similar findings were also reported from different parts of Ethiopia like 47.5% from Bahir Dar [20], 45.7% from Gondar [19], and 44% from Addis Ababa [22].

The predominance of *Escherichia coli* in this and other studies is attributed to it’s a commensal of the bowel, and this owing to the fact that commensals of the intestine are more involved in the UTI due to its proximity to the genito-urinary area anatomically [44]. Besides, *Escherichia coli* is also considered uropathogenic due to some virulence factors (the P-fimbria and S-fimbria adhesions) specific for colonization and invasion of the urinary epithelium [45]. However, our result contradicts with a study from Minna, Niger state where *Klebsiella pneumoniae* showed the highest frequency of occurrence (39.1%) [35]. On contrary, *Klebsiella pneumoniae* was the third frequently observed isolates (16.1%) next to the *Proteus* spp in our case. The difference observed might be due to the endemicity of the isolate in the hospital, climatic, and geographic variation of the study sites [46].

Antimicrobial resistance among uropathogens to commonly used antibiotics is become increasing that left clinicians with very few choices of drugs for the treatment of UTI [4]. Likely, more than half of the isolated uropathogens in the present study showed resistance to ampicillin, amoxicillin-clavulanate, co-trimoxazole, and nalixidic acid. However, our study found that more than 70% of the isolated uropathogens were susceptible to amikacin, nitrofurantoin, gentamicin, and chloramphenicol. The highest susceptibility pattern for the aforementioned drug might be due to the drugs were less likely prescribed or rarely purchased without a prescription in the study area [24]. However, a high rate of resistance might be due to the earlier frequent exposure of the isolate to the above mentioned drugs, and/or the drugs might be used frequently for empiric therapy, or it might be used irrationally which fosters the occurrence of drug resistance [14, 46].

The majority of *Escherichia coli* (≥ 70%) were susceptible to different classes of antimicrobial agents in our study. A study conducted in Dire Dawa, Ethiopia [19], also reported similar rate (≥ 77%) of *Escherichia coli* susceptibility to each gentamicin, chloramphenicol, and ciprofloxacin. However, a study done in Kanchipuram, India [38], indicated that only 40% of *Escherichia coli* showed susceptibility to each amikacin and nitrofurantoin. Such variation might happen due to the existence of a resistant strain of *Escherichia coli* or could be due to the prior antibiotic usage and self-medication [4, 21].

Different studies in Ethiopia reported high rates of ampicillin-resistant *Escherichia coli* like in Gondar (100%) [19], and Dire Dewa (77.8%) [21]. However, the findings from both Gondar and Dire Dewa were by far higher than our reports (50.2%). Comparable to our report, the study in Nairobi, Kenya also indicated that 50% ampicillin resistant *Escherichia coli*. [14]. However, the same study in Nairobi, Kenya [14] also reported that the complete susceptibility of *Escherichia coli* to amoxicillin-clavulanate where high rate (42.9%) of amoxicillin-clavulanate resistant *Escherichia coli* recorded in our case. The rampant and irrational use [14, 24] of ampicillin, and amoxicillin-clavulanate in Ethiopia for a long time might contribute to the development of resistant *Escherichia coli.*

In the present study, *Klebsiella pneumoniae* conferred the highest rate of susceptibility (100%) to each of amikacin, chloramphenicol, and nitrofurantoin which is concurrent with the reports from India [38], Nairobi, Kenya [14], and Gondar, Ethiopia. [19] However, it was in contrast with the study reported from Dire Dewa, Ethiopia [21], where a low rate of susceptibility to chloramphenicol (33.3%) and a high rate of resistance (100%) to nitrofurantoin observed. The variation among the reports might be due to the existence of resistance strain microorganisms or the use of such drugs inappropriately in the study area [14]. Ceftriaxone showed higher activity towards *Pseudomonas aeruginosa* than any other antimicrobial in the present study while ciprofloxacin was effective for only 50% of the pathogen which was in line with the study done in Dire Dewa, Ethiopia [21]. The 100% susceptibility of *Proteus* spp*.* and *Staphylococcus aureus* in our study to amikacin and nitrofurantoin was also comparable with the finding from Harar, Ethiopia [47].

In general, the majority of the isolates in our study are susceptible to less frequently used amikacin, gentamicin, and nitrofurantoin while they are less susceptible to frequently used ampicillin, amoxicillin-clavulanate, and co-trimoxazole.

Our study also revealed that multidrug resistance (i.e., resistance to at least three antimicrobial agents belonging to structurally different classes of antimicrobials) was recorded in 16(51.6%) of isolated bacterial uropathogens. Fortunately, our finding is lower than the previous studies conducted in different parts of Ethiopia, that indicate 100%, 95%, and 74% MDR in Dire Dewa [21], Gondar [19], and Addis Ababa [22], Ethiopia respectively. And MDR was found to be very high among frequently used antibiotics [24]. Despite the highest prevalence of *Escherichia coli* in our study, high prevalence of MDR was observed among *Pseudomonas aeruginosa* (100%), *Proteus* spp.(71.4%), and *Klebsiella pneumoniae* (60%). An inappropriate antibiotic use by patients, inappropriate prescription for empiric therapy by physicians, inappropriate use of antibiotics for nonhuman purposes such as in raising livestock and animal muscle growing activities, and lack of appropriate infection control strategies accelarates a rate of isolate resistance to available antibiotics [7, 19, 24, 46, 48].

Our findings indicated that a high proportion of UTI was recorded among the age group of 23–27 years (45.2%). Likely, a study in Gondar, Ethiopia [19] revealed the high incidence of UTI (12.5%) among patients in the age group of 26–30 years. Moreover, a study conducted in southeastern Nigeria reported a high proportion (61.5%) of UTI within the same age group [44]. This might be due to women in this age group are more sexually active which might predispose them to UTI. However, a high proportion of UTI (100%) was reported among the age group of (40–49 years) from a study done in Minna, Niger state [35]. A higher proportion of UTI was also associated with anatomic or functional defects, such as incontinence, post-void residual urine, cystocele, or relative lack of estrogen accompanied with a loss of normal lactobacillus-dominant vaginal flora, elevated vaginal pH, increased intraorbital colonization with *Escherichia coli* [49].

In addition, our study participants with a history of UTI, catheterization, and prior Obstetric and Gynecologic Surgery showed a higher proportion of significant bacteriuria than their counterparts. Similar findings were reported in Ethiopia and other parts of the world [19, 21, 50–52]. These might be happened due to the presence of resistant strains, prior use of inappropriate treatment, or irrational use of prescribed antimicrobials [24], or frequent and long exposure to catheter use or its improper insertion [19].

Although our study explored the prevalence of UTI, and antibiotic susceptibility of isolated bacteria from urine, it is not with out limitations. Our study did not consider the type of antibiotic the participants used before. The inability to address the clinical outcome of those pregnant women with uropathogens and addressing only bacterial pathogens were the other limitation. Moreover, the inability to identify some bacterial isolates into serotypes and our inability to determine the mechanisms of antimicrobial resistance due to a lack of resources were the major limitation of the present study. And we did not ascertain whether resistance to antibiotics in the in-vitro study does indeed predict a higher rate of treatment failure.

## Conclusion

In general, our study showed that the overall prevalence of UTI was high, 15.5%. The majority of isolated bacterial uropathogens were Gram-negative bacteria, and *Escherichia coli* was the most frequent isolate followed by *Proteus* spp., *Klebsiella pneumoniae*, *Staphylococcus aureus*, and *Pseudomonas aeruginosa*. Majority of the isolates were susceptible to Amikacin, nitrofurantoin, and gentamicin although a high rate of resistance was observed to ampicillin, amoxicillin-clavulanate, and cotrimoxazole from all isolated bacterial uropathogens. More than half of the isolated bacteria had multiple drug-resistant features. Therefore, periodic and continuous urine culture for screening and diagnosis is mandatory to reduce the consequence of UTI and multidrug resistance bacteria in pregnancy.

## Supplementary Information

**Additional file 1.** Semi-structured questionnaire.

**Additional file 2.** Antimicrobial susceptibility test interpretation.

## Data Availability

The datasets generated and analyzed during the current study are available from the corresponding author on a reasonable request.
