# Supplementary material for: Prevalence of bacterial uropathogens and their antimicrobial susceptibility patterns among pregnant women in Eastern Ethiopia: hospital-based cross-sectional study
Source: BMC Womens Health. 2021 Aug 7;21:291. doi: 10.1186/s12905-021-01439-6 (PMC8348837; doi:10.1186/s12905-021-01439-6)
Supplement: Supplementary file 1 — Additional file 1. Semi-structured questionnaire. [file 12905_2021_1439_MOESM1_ESM.docx]

**Additional file 1:** **Semi-structured questionnaire**

Questionnaire for socio demographic and clinical data of UTI among pregnant women Attending ANC in Hiwot Fana Specialized University Hospital, Ethiopia

**Date: ______________________**

**Code number: ______________**

**Card number: ______________**

**Address**

**Zone________________**

**Age _________________ Telephone number_______________________**

**I. Socio demographic characteristics**

1). Residence A) Urban B) Rural

2) Educational level

A) No formal Education C) Primary school (1-8)

B) Read and write D) High school (9-12) E) > 12grade

3. Marital status?

A) Married B) Divorced C) Widowed

4. Occupation?

A) Unemployed D) Farmer

B) Employee E) student

C) Merchant (Trader)

**II. Clinical finding**

1. Presence of burning sensation or pain during urination Yes  No
2. Presence of blood in the urine Yes No 
3. Frequency ( >7 urination per day and > 3 during the night time) Yes No
4. Urgency Yes No
5. Incomplete voiding Yes No

1. Presence of one or more of the following symptoms

Fever flank pain Rigors

Nausea Vomiting Loin pain or tenderness

1. Does the patient is symptomatic Yes No

**III. Risk factor assessment**

1. History of Obstetric and Gyanacologic surgery Yes No
2. Number of pregnancy---------------
3. Parity A) Nullipara B) primipara(one) C) Multipara
4. Gestational age

First trimester Second trimester Third trimester

1. History of urinary tract infection Yes No
2. History of use of catheterization Yes No
3. Co -morbidity A) DM B) HIV/AIDS C) Renal disease
4. History of Antibiotic use without prescription A) Yes B) No

**III) Culture and AST result report form**

**Age__________ Participant code ________________**

| Antimicrobial Susceptibility Test for the study participants with positive urine culture | | | | | | | |
| --- | --- | --- | --- | --- | --- | --- | --- |
| **Isolated Pathogen** | **Microbiological Urine Test Result** | | | | | | |
|  | **Antimicrobial Disk** | **Susceptibility Test*** | | | | | |
|  |  | **Zone of Inhibition** | **Antimicrobial Profile** | | | | |
|  |  |  | **S** | **I** | | **R** | |
| 1. ___________ 2. ___________ | Amoxicillin+clavulanate (30µg) |  |  | |  | |  |
|  | Ampicillin (10µg) |  |  | |  | |  |
|  | Norfloxacine (10µg) |  |  | |  | |  |
|  | Ciprofloxacin (5µg) |  |  | |  | |  |
|  | Gentamicin (10µg) |  |  | |  | |  |
|  | Amikacin (10µg) |  |  | |  | |  |
|  | Ceftriaxone (30µg) |  |  | |  | |  |
|  | Ceftazidime (30µg) |  |  | |  | |  |
|  | Cotrimoxazole (1.25/23.75µg) |  |  | |  | |  |
|  | Chloramphenicol (30µg) |  |  | |  | |  |
|  | Nalixidic Acid (30µg) |  |  | |  | |  |
|  | Nitrofurantoin(300µg) |  |  | |  | |  |
| Name of sample Examiner _______________ Signature _______________  Date _______________ | | | | | | | |

*****interpreted based on the standards adopted from CLSI guideline, 2017; S=Susceptible, I=Intermediate, R=Resistant
