# Supplementary material for: Prevalence of bacterial uropathogens and their antimicrobial susceptibility patterns among pregnant women in Eastern Ethiopia: hospital-based cross-sectional study
Source: BMC Womens Health. 2021 Aug 7;21:291. doi: 10.1186/s12905-021-01439-6 (PMC8348837; doi:10.1186/s12905-021-01439-6)
Supplement: Supplementary file 2 — Additional file 2. Antimicrobial susceptibility test interpretation. [file 12905_2021_1439_MOESM2_ESM.docx]

# Additional file 2: Antimicrobial susceptibility test interpretation

|  | | **Zone diameter to nearest whole mm** | | | |
| --- | --- | --- | --- | --- | --- |
| **No** | **Antibiotic** | **Disk content** | **Susceptible (S)** | **Intermediate (I)** | **Resistant (R)** |
|  |  |  |  |  |  |
| 1 | Amoxicillin+clavulanate | 30 µg | ≥ 17 | 14- 16 | < 13 |
| 2 | Ampicillin | 10µg | ≥ 17 | 14 - 16 | ≤ 13 |
| 3 | Ceftazidime | 30 µg | ≥ 21 | 18 - 20 | ≤ 17 |
| 4 | Ceftriaxone | 30 µg | ≥ 23 | 20 - 22 | ≤ 19 |
| 5 | Chloramphenicol | 30 µg | ≥ 18 | 13 - 17 | ≤ 12 |
| 6 | Norfloxacin | 10µg | >21 | 16-20 | <15 |
| 7 | Ciprofloxacin | 5µg | ≥ 21 | 16 - 20 | ≤ 15 |
| 8 | Cotrimoxazole | 25µg | ≥ 16 | 11 - 15 | ≤10 |
| 9 | Gentamicin | 10 µg | ≥15 | 13 - 14 | ≤ 12 |
| 10 | Amikacin | 10 µg | ≥17 | 15 - 16 | ≤ 14 |
| 11 | Nitrofurantoin | 300 µg | ≥ 17 | 15 - 16 | ≤ 14 |
| 12 | Nalixidic Acid | 30 µg | ≥ 19 | 14 - 18 | ≤ 13 |
|  |  |  |  |  |  |

Source: CLSI, 2017
